# Facilitators and barriers for implementing screening brief intervention and referral for health promotion in a rural hospital in Alberta: using consolidated framework for implementation research

**DOI:** 10.1186/s12913-024-10676-y

**Published:** 2024-02-21

**Authors:** Sharon S. Mah, Gary F. Teare, Jessica Law, Kamala Adhikari

**Affiliations:** 1 Cancer Prevention and Screening Innovation (CPSI), Public Health Evidence and Innovation (PHEI), Provincial Population & Public Health, Alberta Health Services, Calgary, AB Canada; 2 Department of Community Health Sciences, Cumming School of Medicine, University of Calgary, Calgary, AB Canada

**Keywords:** Screening brief intervention and referral, Health promotion, Cancer prevention

## Abstract

**Background:**

Screening, brief intervention, and referral (SBIR) is an evidence-based, comprehensive health promotion approach commonly implemented to reduce alcohol and substance use. Implementation research on SBIR demonstrate that patients find it acceptable, reduces hospital costs, and it is effective. However, SBIR implementation in hospital settings for multiple risk factors (fruit and vegetable consumption, physical activity, alcohol and tobacco use) is still emergent. More evidence is needed to guide SBIR implementation for multiple risk factors in hospital settings.

**Objective:**

To explore the facilitators and barriers of SBIR implementation in a rural hospital using the Consolidated Framework for Implementation Research (CFIR).

**Methods:**

We conducted a descriptive qualitative investigation consisting of both inductive and deductive analyses. We conducted virtual, semi-structured interviews, guided by the CFIR framework. All interviews were audio-recorded, and transcribed verbatim. NVivo 12 Pro was used to organize and code the raw data.

**Results:**

A total of six key informant semi-structured interviews, ranging from 45 to 60 min, were carried out with members of the implementation support team and clinical implementers. Implementation support members reported that collaborating with health departments facilitated SBIR implementation by helping (a) align health promotion risk factors with existing guidelines; (b) develop training and educational resources for clinicians and patients; and (c) foster leadership buy-in. Conversely, clinical implementers reported several barriers to SBIR implementation including, increased and disrupted workflow due to SBIR-related documentation, a lack of knowledge on patients’ readiness and motivation to change, as well as perceived patient stigma in relation to SBIR risk factors.

**Conclusion:**

The CFIR provided a comprehensive framework to gauge facilitators and barriers relating to SBIR implementation. Our pilot investigation revealed that future SBIR implementation must address organizational, clinical implementer, and patient readiness to implement SBIR at all phases of the implementation process in a hospital.

**Supplementary Information:**

The online version contains supplementary material available at 10.1186/s12913-024-10676-y.

## Introdution

Cancer is the leading cause of death in Canada and globally. It is projected that 43% or 2 in 5 Canadians will be diagnosed with cancer in their life time [1]. In Alberta, the leading cause of death for those ages 35–64 years old was cancer, followed closely by circulatory disease [2]. However, an estimated 40% of cancers are preventable, as they are attributed to modifiable risk factors, including tobacco consumption, alcohol use, physical activity, and low fruit and vegetable consumption [3, 4]. The Canadian Population Attributable Risk of Cancer study conducted in 2015 found that Alberta had 2,780 new cancer cases attributed to tobacco smoking, 994 to low physical activity, 280 to alcohol use, and 891 to low fruit and vegetable consumption [5]. Tobacco use [6, 7], alcohol use [8–10], low physical activity [11–14] and low fruit and vegetable consumption [15–17] are also causally linked to major chronic diseases: diabetes, cardiovascular disease, and chronic obstructive pulmonary disease (COPD) [18]. These chronic diseases and cancers are preventable by changing behavior concerning these risk factors.

Screening, Brief Intervention and Referral (SBIR) is a comprehensive and integrated health promotion intervention that links screening for risk factors with provision of brief advice and referrals to services that support behavior change for patients at elevated risk. In the 1980s SBIR was developed by a consortium of researchers at the World Health Organization (WHO) to screen for alcohol use disorders, followed by subsequent research in 1997 in the United States, Australia, and European countries [19]. The effectiveness of SBIR at reducing alcohol and substance use disorders and decreasing health system cost led to the inclusion of screening for other modifiable risk factors, such as tobacco use [20–22]. Research evidence supports the feasibility of SBIR implementation, its acceptability to patients and providers, and its effectiveness at reducing risk factors related to health behaviors [22–24]. Most research has focused on SBIR for alcohol use in primary care settings or emergency departments, but the implementation and evaluation of SBIR in a wide range of hospital settings and for other modifiable risk factors is still emergent [21].

Hospital settings are ideal for SBIR implementation for several reasons. First, these risk factors account for 22% of healthcare expenditures because they are leading factors of chronic diseases and cancers [25]. Secondly, 33% of patients in hospital beds have conditions related to at least one of the four risk factors [26]. Thirdly, Broyles and her colleagues (2012) have demonstrated that 80% of patients are comfortable with and accept SBIR interventions from nurses. Patients also trust and respond to behavioral health advice from health professionals during healthcare encounters [27].

Understanding the barriers and facilitators to SBIR implementation in a hospital setting is critical to providing guidance for SBIR implementation. Currently, there is little evidence or few best practices to guide SBIR implementation and the integration of health promotion services for multiple risk factors in hospital settings [19, 21]. This lack of evidence has resulted in health promotion initiatives in hospital settings failing to continue beyond the pilot stage [28]. The objective of the research is to examine the barriers and facilitators of SBIR implementation experienced by the project CPSI implementation support team (IST) and the clinical team implementers (CTI) at the rural pilot hospital.

### Pilot hospital implementation setting

The Cancer Prevention and Screening Innovation (CPSI), a unit in Alberta Health Services’ (AHS) Provincial Population and Public Health, developed the health promoting hospitals initiative based on the WHO’s health promoting hospital framework. The CPSI team collaborated with the pilot hospital to test the feasibility of integrating SBIR for tobacco use, alcohol use, low physical activity, and unhealthy diet into the everyday work of healthcare providers.

Between May 2019 and September 2020, 24 healthcare providers (nurses, social workers, and allied public health workers) in 6 units of the pilot hospital (acute care, allied health, addictions and mental health, chronic disease management, home care, and public health) implemented SBIR. SBIR was initially planned for May 2019 to September 2021, but the implementation slowed down in March 2020 due to the COVID pandemic. In September 2020, SBIR was paused indefinitely as clinical staff from the rural hospital and CPSI were redeployed to cover the COVID − 19 pandemic. During the implementation period, 543 patients participated in screening for the four factors, 52.8% were screened at the chronic disease management unit and the rest were from the other five units. Those patients screened as medium or high risk for the factors were: 27% for tobacco use, 35% for alcohol use, 59% for low physical activity, and 78% for insufficient fruit and vegetables. Of those screened medium/high risk, 59–71% received brief advice depending on the risk factor. Lastly, of those screened medium/high risk, 2–29% received referral depending on the risk factor. The detail information about the SBIR implementation processes, coverage of the SBIR intervention for each risk factors, the effectiveness of the SBIR pilot, and the patient demographics related to the risk factors are published elsewhere [29].

## Methods

### Study design

We carried out a qualitative inquiry based on the interpretivist approach, pragmatism, using an abductive approach to thematic analysis to evaluate the barriers and facilitators of SBIR implementation in a hospital setting. This approach combines both inductive and deductive analyses to gauge and analyze the implementation teams’ unique experiences and learnings within the theoretical context of the Consolidated Framework for Implementation Research (CFIR). The study was conducted after the indefinite pause of project implementation due to COVID-19.

### Study participants

In October 2021, we invited clinical team implementers (CTI) and project implementation support team members (IST) to participate in the current qualitative study. A study invitation was sent by email to all implementation team members. We recruited participants through a combination of convenience and snowball sampling. COVID-19 was a main barrier for recruitment as clinical staff were redeployed to respond to the COVID − 19 pandemic. We used the snowball method to reach as many clinicians as possible since we could not determine who from the original pilot units were available or still located at the hospital. A total of six semi-structured, in-depth interviews were carried out with two clinical implementers and four support members. The clinicians included two registered female nurses from the pilot hospital. The first nurse was the super-user for SBIR implementation. She was also the facilitator who mentored other clinicians implementing SBIR in the six units at the hospital for the duration of the project. She was the on-ground day-to-day contact for all the clinical implementers and CPSI team. She had the experience of all implementation processes, barriers, and facilitators across all the units. The second nurse’s primary role was administrating vaccines in the public health unit, and she completed SBIR with her patients. The implementation support participants consisted of two managers, a program coordinator, and a program evaluator from CPSI, AHS, who had engaged all units to co-design and monitor the SBIR implementation process across units. During the interviews, we encouraged study participants (except the public health nurse, who was asked to share information related to her unit) to reflect on the SBIR work across the units. Most interviews lasted between 45 and 60 min, with one participant completing the interview in 15 min.

Written informed consent procedures were virtually completed. The current study received approval from our institution’s research ethics board.

### Study procedures

From October to November 2021, all participants took part in a virtual, semi-structured in-depth interview. Interviews were conducted by a study investigator with mixed-methods research expertise (SM), who had not previously worked with the interviewed team members. Two investigators prepared interview guides with a standardized set of questions and discussion-stimulating probes informed by the CFIR framework (SM, KA). All interviews were conducted, audio recorded, and transcribed over the Zoom videoconferencing platform. As a quality control measure, all transcripts were reviewed and compared against their original audio file to verify accuracy and completeness. After which, audio files were digitally discarded. Participants’ confidentiality and privacy was maintained by de-identifying all transcripts and redacting any personally identifying information. Each interviewee was also assigned a reference number.

### Interview guide

Informed by the CFIR, two interview guides were prepared for clinical implementers and project support team members. The CFIR provides a comprehensive framework for identifying and classifying barriers and facilitators that impact program implementation in healthcare settings [30]. We took a systematic approach to developing two, participant-specific interview guides, recognizing the distinct role clinical and project support teams played throughout the pilot implementation process. As such certain CFIR domains were more applicable to specific team members. For example, only IST participants were asked **Process–*planning, engaging*** questions since they were involved in the related tasks. Questions regarding **Characteristics of the Individuals–*stage of change and self-efficacy*** were only asked of clinical implementers because they integrated SBIR into their practice. A breakdown of interview question by CFIR domain is provided in Supplementary Table 1. ***Data analysis***.

We adapted Thompson’s (2022) abductive or hybrid approach to thematic analysis, combining an initial bottom-up, inductive thematic analysis of interview transcripts, with a subsequent deductive analysis in which interviews were situated and contextualized within the broader CFIR framework [31]. All coding decisions and analytic direction were discussed among the study team.

Initially, an inductively driven analysis was performed, using Clarke and Braun’s (2006) six-step process: data familiarization, generating initial codes, searching for themes, defining, and naming themes, and producing a report. Inductive thematic analysis allows us to examine the unique perspectives, highlight similarities and differences, and generate unanticipated insights [32]. First, the primary investigator carried out a comprehensive and in-depth scan of all interview transcripts, noting emerging trends and patterns in brief analytic and reflexive memos. Second, an initial matrix of descriptive codes was generated from the transcripts of our first three interview, capturing patterns and commonalities observed in the raw data. This matrix served as a starting point for the coding of subsequent interviews. The codes were iteratively refined to ensure they were distinct and non-repetitive. Third, the team analyzed connections between and across codes and abstracted a set of themes and sub-themes, which were subsequentially tested for referential adequacy within the raw data. Finally, in line with the research question, themes were organized into two overarching components, facilitators and barriers.

Subsequently, a deductive analysis was carried out, whereby the five CFIR domains and 28 constructs were used to organize and interpret emerging themes and prepare a narrative synthesis of our qualitative findings. The themes were deductively mapped back to the excel spreadsheet containing the CFIR domains, constructs, and initial questions. To ensure that barrier and facilitator themes were mapped to the appropriate CFIR domains and constructs, all investigators reviewed and refined the synthesized table of results. Representative quotes were reported for each identified theme. Findings were organized by facilitators and barriers, and specific CFIR domains/constructs. This analysis allowed us to provide a substantiative account (thick description) of various facilitators and barriers as we were able situate our emerging themes within the broader contextual determinants of implementation. All data organization, coding, and analyses were carried out in NVivo 12 Pro.

## Results

Our inductive analysis revealed barriers and facilitators to SBIR implementation in six contexts: the SBIR tool, patients, clinical implementors, the pilot hospital, the Alberta health care system, and the broader community. Implementation support team participants primarily perceived facilitators to SBIR implementation while clinical implementors largely perceived barriers to implementation. This may reflect each group’s unique roles and capacity of engagement throughout the implementation process (Supplementary Table 2).

### Key facilitators to SBIR implementation

At the health system level, the key facilitator to SBIR implementation was related to ***planning and engagement***, as outlined in Table 1. Prior to SBIR implementation, the support team built formative and collaborative relationships with relevant clinical departments within Alberta Health Services (AHS). These relationships helped to align the intervention with Canadian guidelines and AHS’ health promotion and disease prevention standards on fruit and vegetable consumption, tobacco use, alcohol use, and physical activity. This enabled the development and evaluation of the SBIR intervention.

**Table 1:** Key facilitators to SBIR implementation

CFIR Domain - Construct	Primary Theme	Sub Themes	Quotes
**Process–** ***Planning***	**IST**: Planning process in AHS (systems level) Planning at pilot site Planning for patients’ information needs Operational plans needed to support SBIR implementation	• Collaboration with AHS teams to align risk factor screening with Canadian guidelines; align AHS health practices, standards, evidence, and ethics for risk factors; develop process to acquire data from Cancer Epidemiology and Prevention Research dept • Promotional material facilitate understanding - professional looking and branding facilitate buy-in • Printed health information easy to use • Evaluation of SBIR was supported by evaluation expertise; easy to understand scripts for clinical workers; training materials for users; funding a dedicated program facilitator	“There are departments within Alberta Health Services that own a risk factor, so Nutrition Services owns healthy eating, that’s the way they would see it. So, [we worked] with leadership in departments… to tailor the questions in a way that did not compete or contradict any of the in-depth screening that they do… [it is] very important for that alignment.” (Interview 1)
***Engaging***	**IST**: Engaging with hospital teams Facilitator engaging with CTI Engagement with hospital management	• Engaging with facilitator using regular touch base conversations facilitate adaptations of the SBIR; on the ground facility understanding; time to train users; facilitate user comfort and comprehension of the SBIR tool - knowledge and awareness risk factors; willingness of CTI to participate and be involved • CTI champion facilitates usage of the SBIR tool - connected other clinical staff • Feedback reports used to understand patient referral- Decision making around how to structure report to meet needs of hospital unit; communication and coordination for sustainability of the project; seeing the big picture or goal of the project; supportive team provided on site help	“Hearing the response from the facilities,… when they receive those reports.… they were well received.… They were very respectfully put [together]. There was a back-and-forth process, so the facility co-designed that feedback loop.…. And because of that we felt confident that it would… keep the wind in the sail for the clinicians.” (Interview 1)
**Inner Setting–** ***Readiness for***	**IST**: AHS health system readiness for change– *leadership engagement to develop coordination between depts* Pilot site readiness: 1. Available resources needed to implement SBIR and foster buy-in 2. Access to knowledge and information to create confidence 3. IST assessed leadership readiness as being prepared for SBIR implementation	• Collaboration with AHS teams to align components / departments in system: align SBIR with Canadian guidelines; align with AHS departments on risk factor question: align with internal AHS screening guidelines; aligning health practices, standards, evidence, and ethics. • Funds for human resources; existing relationships with staff and community; meeting end-users needs (1-page SBIR screening tool to fit clinician time); develop SBIR workflow with clinic • Developed scripts for CTI to reduce fear of using the tool; SBIR training for CTI • Existing knowledge, skills, and leadership buy-in, co-designed feedback reports	“We put those scripts together that would give a guideline… because that was something that we did run into, ‘I don’t know how to talk about this’. ‘I don’t know how to discuss tobacco’ or ‘I don’t know how to discuss alcohol’. [It] really made a difference… because it took away that fear.” (Interview 3) “I think that the written materials were very comprehensive. The training always helps. The one pager and other promotional materials [were]…very important for the clinicians, and for the patients as well…” (Interview 1)
***implementation***			
***Available*** **resources**			
***Access to knowledge and***			
***information***			
***Leadership readiness***			
*Diverging Perspectives of Facilitator and Barrier (opposing views between support and clinical team on implementation climate)			
***Implementation climate***	**IST (Facilitator)**:	• Implementation driven by managerial decision.	“I do think that having more leadership involvement would be useful for swaying people’s perspectives on getting a little bit more buy-in… [SBIR implementation facilitator] was very invested.… It was good because she had a great relationship with a lot of the other nurses, so she was able to get them on board.” (Interview 5)
	**CTI (Barrier)**:	• Implementation climate was driven by managers: This was seen as a top-down process coming from managers regardless of clinical staff’s own views.	“I would need buy-in from… all managers, at the table, not on a Zoom conference. I had one manager… who is [the] worst… I’ve sent you 8 million emails about this. So of course, she’s not promoting [SBIR] and checking with her staff to see if they’re doing [SBIR].” (Interview 4)

SBIR: Screening brief intervention and referral

CFIR: Consolidated Framework for Implementation Research

IST: Implementation support team

AHS: Alberta Health Services

CTI: Clinical team implementers

At the hospital level, the implementation support team customized PowerPoint presentations and promotional materials to brand the SBIR intervention (***readiness for implementation***, ***leadership engagement***). These resources facilitated managers’ understanding and buy-in. To ensure successful SBIR implementation, feedback reports were co-designed with unit managers to support decision making (***Readiness*- leadership*access to knowledge and information***).

The support team developed flexible drop-in style training sessions, customized the SBIR to clinicians’ workflow, and created scripts to support clinicians’ confidence in conducting SBIR with their patients (***Readiness– access to knowledge and information***). As well, the support team funded a dedicated implementation facilitator (**Readiness–*available resources*)** at the pilot hospital and trained SBIR super-users to support clinical implementers’ information needs and confidence during SBIR with patients **(*knowledge and self-efficacy*)**. The support team perceived that training would increase clinical implementers’ understanding of all SBIR risk factors and their impact on patient health. The support team also perceived that training would increase clinical implementers’ motivation and confidence to apply SBIR within their practice **(Readiness–*available resources*)**.

The support team enabled the SBIR intervention to be operationalized by developing context-specific resources for all stages of the SBIR intervention: feedback reports, funding a dedicated SBIR implementation facilitator, training, and resources for clinical staff to implement SBIR with patients, and patient risk factor pamphlets **(Readiness–*access to information*)**.

### Key b to SBIR implementation

Clinical implementers faced several challenges with integrating SBIR plans, training, resources, and tools into their clinical practice, as summarized in Table 2. In particular, clinicians noted barriers related to ***adaptability and design quality and packaging***. First, clinicians reported that the screening questions did not fit with the patient’s appointment purpose. Second, clinicians indicated the SBIR paper screening form made it difficult to manage patient information: potential for errors in data transfer; restricted information flow (using fax machine); incomplete screening due to user error; and possible breech of patient confidentiality and privacy (faxing to a unit outside of the hospital). Third, the SBIR intervention impacted clinicians’ workflow and workload by increasing the amount of time they needed to spend with the patient. Clinicians reported that they often referred to paper-based educational materials because they forgot the meaning of the risk assessment score for each factor. Despite the support teams’ efforts to tailor the SBIR intervention to clinicians’ workflow, the data collection increased clinicians’ existing workload and interrupted their workflow.

**Table 2:** Key barriers to SBIR implementation

CFIR Domain - Construct	Primary Theme	Sub Themes	Quotes
**Intervention Characteristic**– ***Adaptability*** ***Design quality & packaging***	**CTI**: Participants perceived SBIR paper form could not be adapted to patients or local needs: Patients were overwhelmed by the amount of brochure information **IST**: Paper format increased workload **CTI**: SBIR paper format interrupted workflow and limited understanding of risk factor assessment	• Questions did not always fit with the main reason for patients’ appointments/visits; referral resources for healthy eating and physical activity not found in patients’ community • SBIR form did not prioritize which factors should be focused on, so nurses gave information for all medium to high risk factors. • Easy to make errors for data transfer; hinder information flow; incomplete due to user error; patient confidentiality and privacy • Risk level calculation algorithm for factors in SBIR form was not obvious or easily understood - needed continue referencing to paper source to understand; limited number of health factors covered	“Some of the questions I thought were good conversation starters, [but] for lots of people they wondered, ‘why is this important, how many fruits and vegetables I eat when I’m here for my addictions appointment?’” (Interview 4) “[CTI] didn’t like it, especially to scan it back [to IST], and then evaluate the results that needed to be completed in a specific way.…I think it created some extra problems… If you accidentally put a mark somewhere,…then it would show up incorrectly in our system.” (Interview 5) If [patients] end up taking up the whole 15 min because there’s a lot of questions about the vaccine, then there was no time left to go through those questions… so your intervention was just really time consuming” (Interview 6)
**Outer Setting**– ***Patient’s needs & resources***	**CTI**: Patients’ needs and resources not met CTI did not prioritize SBIR factors Community lacked resources for patients Health system barriers to meeting patient needs Patients’ knowledge and beliefs Relationship between provider and patients	• Patients found brochures and information from SBIR overwhelming; timely resources are needed– lengthy referral wait time • Insufficient clinical programs or resources to support patients when they are ready to change • Difficult referral pathway after SBIR questionnaire was completed - long wait time reduces patients’ motivation to change; the referral pathway focuses on disease treatment not on prevention; lack of resources to support patients’ readiness to change • Patients’ health choices are based on health inequities: inability to understand scientific studies and assess risk; lack of early health prevention education; lack of health behavior understanding. • Patients desire for immediate results from initial health choice - “an all or nothing approach” that prevents behavior change and choices; choices are limited due to poverty and convenience; lack of understanding of food choices and health problems; need easy win solutions for changes to behavior • Lack of trust in the clinicians– past negative experiences with clinicians can create hesitancy to disclose alcohol and tobacco usage	“Maybe you’re motivated for a minute but if we make you wait six weeks, [you’re] not really interested in going for a walk or… I think we must catch people when they’re ready.” (Interview 4) “I feel community stakeholders would have been better… places to refer [patients] for the dietitian. If they want to increase their exercise, it would have been nice to have some type of input on community resources and where they could go to increase activities.” (Interview 6) “We spend way too much time on tertiary care and not so much on preventative and primary… I think, we’re trying to put out fires.” (Interview 4) “I think [people] just always want that immediate gratification. If we go for a walk one day, we want to think that we’ll get a washboard stomach, and it doesn’t quite work that way. (Interview 4).
**Characteristics of the Individuals–** ***Knowledge and beliefs***	**CTI**: CTI were hesitant to implement SBIR Patients’ knowledge / beliefs about health and hesitancy to change Pilot site management hesitancy	• Risk factor screening questions to patients deemed too personal and not within provider role; small community means clinician and patient are neighbors; perception that SBIR would not make a health difference (provider see patient in late stage of health decline; provider see patients choices influenced by other factors like poverty and cannot make changes); patients lack interest in the questions • Desire for immediate results from initial health choice; inability to understand scientific studies and assess risk; lack of early health prevention education; lack of health behavior understanding - All or nothing approach that prevents behavior change and choices; choose convenience over healthy options; lack understanding of food choices and health problems • Patients’ late stage of health decline and SBIR is too late	“You absolutely need to teach people to be comfortable with the questions. You need to teach them to know how to handle the answers,… oh you’re only having one glass of wine at night, which is good, right?…I think in healthcare where we go wrong, is that kind of high and mighty attitude.” Interview 4

SBIR: Screening brief intervention and referral

CFIR: Consolidated Framework for Implementation Research

IST: Implementation support team

AHS: Alberta Health Services

CTI: Clinical team implementers

Clinicians noted barriers related to ***patients’ needs and resources***. First, clinician’s felt that by providing information brochures to all medium and high-risk scoring patients, they did not consider the patients’ readiness to change. Second, clinicians indicated that referral wait times were lengthy for risk factor behavior change resources. They perceived that patients’ readiness to change could be supported after SBIR with immediate access to resources within the community, especially for physical activity and fruit and vegetable intake. Third, clinicians perceived that the difficult referral pathways and AHS health system barriers were reflective of broader challenges with the healthcare system’s focus on disease treatment rather than prevention. Clinician felt that healthcare providers often see patients at later stages of health decline rather than preventing disease. During SBIR implementation, clinicians discovered that they had limited knowledge on supporting patient through behavior changes and there were scarce ***patients’ resources*** on physical activity and nutrition.

Clinicians perceived barriers related to ***patients’ knowledge and beliefs***, reporting they often encountered patients expecting immediate health benefits from short term behavior changes. They recognized that patient’s choices were based on convenience and felt that patients lacked health literacy. Clinicians indicated that they often did not know how to address patients’ beliefs on instant behavior change or immediate health benefits, and this resulted in their low motivation to implement SBIR. While clinicians were provided SBIR training, they lacked the experience and clinical training to address patients’ questions regarding the modifiable risk factors. Clinicians indicated that healthcare providers tend to judge “good” and “bad” behaviors rather than recognizing that patients’ process to understanding and changing risk factor behavior requires small steps or gradual behavioral change. Clinical implementers reported that executing the referral process proved difficult due to the lack of trust between providers. Perceived challenges to ***executing***, ***reflecting***, and ***evaluating*** can be found in the Table 1.

### Diverging perspectives on facilitators and barriers

We noted tensions between how participants defined barriers and facilitators. For example, the support team and clinical implementers held opposing views on ***implementation climate.*** For the support team, management buy-in and leadership involvement was a driving factor to sway clinician hesitancy to implement.

The support team perceived management leadership and project champions were key to facilitating implementation. They also perceived that identification of super-users would facilitate implementation by developing workflow pathways. Clinicians, however, felt that all managers and super users did not buy into SBIR. As a result, they could not influence clinicians’ hesitancy or their attitudes towards SBIR.

The different perspectives on management, super-users, and champions could be attributed to the role and position that the support team and clinical implementers played in the SBIR implementation, which affected their ability to observe SBIR in action.

## Discussion

The clinical implementers primarily reported barriers and the implementation support team primarily reported facilitators to implementing SBIR. Tackling the barriers to SBIR implementation is of paramount importance to effectively develop better plans to support future SBIR implementation in hospital settings.

The support team and clinical implementers both encountered challenges with the paper format of the SBIR (***design***), which interrupted clinicians’ workflow and data management. Other studies have indicated that when SBIR is integrated into the electronic health records (EHR), workflow barriers were removed: audio-visual aids and systemized reminders facilitated innovation implementation to support workflow [21, 33]. Paper SBIR forms have been negatively perceived as an unsustainable innovation because it exists outside of the EHR as a separate work process [34]. Comparative studies of the effects of paper-based and EHR processes on healthcare provider work practices have demonstrated the value of EHR to significantly improve the efficiency of clinicians’ use of time [35]. Using EHR can streamline clinicians’ workflow. It also removes the need to manually fax patients’ information from one site to another as all EHR are linked together. EHR align health systems operation, workflow, and enables healthcare providers to intervene at earlier stages of patients’ health [36, 37]. However, EHR also create vast changes to the healthcare system that requires technological interoperability, stakeholder involvement across the healthcare system, and significant government investment [38]. As well, it is important to understand the social context for implementation will vary depending on the care setting [39]. EHR are not a panacea to public health challenges, but they can be an effective tool when designed with end-users in mind.

Clinicians reported barriers to SBIR implementation related to their own, and their patients’ ***knowledge / beliefs*** and ***self-efficacy***, which led to a hesitancy to complete SBIR with patients. Other studies have also found healthcare providers often lacked sufficient SBIR related knowledge and SBIR training to reduce the stigma associated with substance use [33, 40, 41]. Evidence indicates that formal training in SBIR for clinicians facilitates ***knowledge*** and ***self-efficacy*** in SBIR [42]. However, educational programs for SBIR and substance use remain inadequate and limited with a predominate focus on physicians [43]. In the current post-pandemic climate, future SBIR implementation will need to consider co-designing SBIR education for nurses, provide flexible training schedules, and comprehensive education materials that are also time sensitive [41, 44].

Clinicians perceived significant barriers for patients **(*patients’ needs and resources*)**, particularly patients’ lack of risk factor knowledge, readiness to change, and the stigma associated with disclosing alcohol use. Other studies have also found patients often lack knowledge about risk factors, the skills and motivation to change their behavior [40]. Clinicians and patients’ perceptions of stigma around alcohol [45, 46]; weight- healthy eating [47–49]; and tobacco [50] impacts patients’ decision to seek medical care [51] and contributes to adverse health outcomes [52]. Stigma related to these risk factors have been documented in the literature, which have created hesitancy in clinicians and patients [53]. Future SBIR implementation will need to co-design patient-centered communication strategies in SBIR with patients [54–56] and train clinicians to provide nonjudgmental screening and brief intervention. Some destigmatizing and nonjudgmental approaches involve understanding the terminologies used by communities, demonstrating understanding and acceptance [57] and disentangling the patients’ behavior from their identity [58].

Studies have also found that patients lack sufficient resources and programs in their communities to support their risk factor related behavior change [41, 59]. Strategies to mitigate the paucity of support services in communities may include electronic health (eHealth) solutions provided through websites (programs, social networks), mobile devices (smart phones, tablets), and digital technologies (apps and tracking devices). eHealth patient resources for physical activity [60], physical activity and healthy eating [61], alcohol use [62], and smoking cessation [63] demonstrate effectiveness as standalone applications or in combination with face-to-face clinical support. However, just as there are health inequities, there are also communication and technology inequities. eHealth solutions must be designed and implemented with underserved populations in mind, which means eHealth solutions may need to create or build patient readiness for digital technology / internet / communication literacy for these types of support services to be usable and effective [64]. If clinicians are expected to utilize eHealth solutions, they will need to be involved in co-designing its utility and incorporation into seamless workflow [60].

Aside from the strategies suggested in the above, many of the barriers found in this study (***networks and communications***, ***culture, implementation climate***, ***readiness for implementation***, ***knowledge and beliefs***, ***self-efficacy***) can be translated as opportunities to assess ***readiness*** [65]. Utilizing a CFIR based readiness assessment provides a systems approach to assessing contextual factors of readiness [65, 66] compared to individual perceptions approach [67]. Miake-Lye et al. (2020) systematic review and content analysis of readiness assessments indicate that nearly all the questions could be mapped back to CFIR. SBIR implementation in Alberta hospital settings would benefit from considering the barriers found in each of the constructs here and assess readiness at different stages of project development and implementation.

### Strength and limitations

This study’s strengths include the use of CFIR as the basis for the development of the semi-structured interview questions, data collection, and analysis. It offers a comprehensive approach to understanding the barriers and facilitators of SBIR implementation. Second, the semi-structured interview foregrounds the participants’ lead and perspectives and ensured our interviews were conversational and organically stimulated discussions around areas of inquiry. Third, our abductive approach to thematic analysis allowed us to not only champion participants’ unique perspectives, but enabled thick description, by contextualizing their experiences within the broader determinants of implementation uptake.

Despite the strength of this research, there are also limitations. First, this is a single site study and only a few of the clinical implementers participated in the interviews. Second, due to the COVID– 19 pandemic, this study took place almost one year after SBIR was indefinitely paused in the pilot hospital. All healthcare teams, including those in the pilot site and CPSI, were responding to COVID-19. Third, the smaller number of clinical participants limits the transferability of study findings to other health contexts. Despite our best efforts to recruit more clinical implementers, employing both convenience and snowball sampling, they were either not available or could not be located after COVID– 19 redeployments. More interviews in the future with clinical implementers would provide greater detail on their ***patients’ needs and resources***, their SBIR ***knowledge and beliefs***, the ***intervention’s adaptability and design***, and diverse perspectives of other barriers and facilitators to SBIR implementation. Fourth, the absence of interviews with patients limited the ability to thoroughly investigate ***patients’ needs and resources*** from their own perspective. Future studies would benefit from taking a patient-centered approach to investigating SBIR implementation to ensure it is acceptable to them. Lastly, future studies will need to document, compare and contrast the differences and similarities of implementing each risk factor from the perspective of the clinicians and patients. Approaches to plan and evaluate SBIR maybe similar, but patients and clinicians’ experiences of each risk factor maybe more nuanced.

## Conclusion

The current study offers unique insights into the facilitators and barriers of implementing a SBIR intervention for multiple risk factors in the hospital setting. The key facilitator of implementation lies with ***planning*** and ***engaging*** partners and stakeholders to ensure that screening questions, advice, and support services were aligned with other departments at AHS. This provided health system alignment around guidelines, questions, and practices. Collaborating with managers and providing SBIR training to clinicians ensured their ***readiness*** to use SBIR. However, clinicians reported several barriers to integrating SBIR into their workflow, including a lack of ***knowledge and self-efficacy*** around supporting patients with behavior change and providing adequate support to meet ***patients’ needs***. Enhancing clinician training to include patient-centered communication, ways to approach patient stigma, and more information resources on the risk factors would improve clinicians and patients SBIR experience. Additionally, the pen-and-paper approach was found to impede clinicians’ workflow and operations. Future implementation studies will explore the delivery of SBIR through an electronic medical record and will integrate barriers/facilitators identified here to improve intervention delivery and clinician readiness.

### Electronic supplementary material

Below is the link to the electronic supplementary material.

Supplementary Material 1: Development of Semi-Structure Questionnaire from CFIR

Supplementary Material 2: Facilitators and barriers of SBIR implementation in hospital settings using the Consolidated Framework for Implementation Research (CFIR)

## Data Availability

The datasets used and/or analyzed during the current study are available from the corresponding author on reasonable request.

## Abbreviations

AHS: Alberta Health Services
CFIR: Consolidated Framework for Implementation Research
CPSI: Cancer Prevention and Screening Innovations
CTI: Clinical team implementers
eHealth: Electronic health
EHR: electronic health record
IST: Implementation support team
SBIR: Screening brief intervention and referral
WHO: World Health Organization
