# Supplementary material for: Facilitators and barriers for implementing screening brief intervention and referral for health promotion in a rural hospital in Alberta: using consolidated framework for implementation research
Source: BMC Health Serv Res. 2024 Feb 21;24:228. doi: 10.1186/s12913-024-10676-y (PMC10882928; doi:10.1186/s12913-024-10676-y)
Supplement: Supplementary file 1 — Supplementary Material 1: Development of Semi-Structure Questionnaire from CFIR [file 12913_2024_10676_MOESM1_ESM.docx]

| **Participant Type** | **CFIR Domains** | **Constructs Covered and # of Questions** | **# Of CFIR**  **Initial Questions** | **# Of CFIR**  **Adapted Semi- structured Questions** |
| --- | --- | --- | --- | --- |
| **IST** | Intervention Characteristics | - Intervention source (1) - Relative advantage (2) - Adaptability (1) - Complexity (1) - Design quality and packaging (1) | 17 | 7 |
|  | Outer Setting | - Patients’ needs and resources (1) |  |  |
|  | Inner Setting | - Readiness for implementation (1) |  |  |
|  | Characteristics of  the individuals | - Knowledge and beliefs (1) |  |  |
|  | Process | - Planning (2) - Engaging (3) - Executing (2) - Reflecting and evaluating (1) |  |  |
| **CTI** | Intervention Characteristics | - Intervention source (1) - Relative advantage (2) - Adaptability (1) - Complexity (1) - Design quality and packaging (1) | 28 | 8 |
|  | Outer Setting | - Patients’ needs and resources (2) |  |  |
|  | Inner Setting | - Networks and communication (1) - Culture (1) - Implementation climate (4) - Readiness for implementation (3) |  |  |
|  | Characteristics of Individuals | - Stage of change (3) - Self-efficacy (1) - Knowledge and beliefs (4) |  |  |
|  | Process | - Executing (2) - Reflecting and evaluating (1) |  |  |

**Supplementary Table 1: Development of Semi-Structure Questionnaire from CFIR**

SBIR: Screening brief intervention and referral

CFIR: Consolidated Framework for Implementation Research
IST: Implementation support team

AHS: Alberta Health Services

CTI: Clinical team implementers
