# Supplementary material for: Facilitators and barriers for implementing screening brief intervention and referral for health promotion in a rural hospital in Alberta: using consolidated framework for implementation research
Source: BMC Health Serv Res. 2024 Feb 21;24:228. doi: 10.1186/s12913-024-10676-y (PMC10882928; doi:10.1186/s12913-024-10676-y)
Supplement: Supplementary file 2 — Supplementary Material 2: Facilitators and barriers of SBIR implementation in hospital settings using the Consolidated Framework for Implementation Research (CFIR) [file 12913_2024_10676_MOESM2_ESM.docx]

**Supplementary Table 2: Facilitators and barriers of SBIR implementation in hospital settings using the Consolidated Framework for Implementation Research (CFIR)**

| **CFIR Domains** | **CFIR Construct** | **Facilitators** | **Barriers** |
| --- | --- | --- | --- |
| **Intervention Characteristic** | **Intervention Source** | Not reported | Not reported |
|  | **Relative Advantage** | **IST:** healthcare system alignment in tackling cancer risk factors | Not reported |
|  | **Adaptability** | **IST:**   - Participants perceived SBIR adaptations was a collaborative process between CPSI and pilot units. - SBIR tool was created with CTI | **CTI:**   - ***SBIR tool could not be adapted to patients or local needs:*** Questions do not always fit with the main reason for patients’ appointments/visits (patient level barriers to the SBIR questions); patient referral resources did not reflect community resources to support patients |
|  | **Complexity** | **IST:**   - ***Complexity of the intervention required AHS department collaboration***: Coordination and collaboration across all sectors of care; deciding on priority areas (may not be in alignment with other departments) | Not reported |
|  | **Design Quality & Packaging** | Not reported | **IST:**   - **SBIR paper format** was a poor design: easy to make errors in data transfer; hinder information flow; incomplete due to user error; risk of violating patient confidentiality and privacy   **CTI:**  **SBIR paper format interrupted workflow:** risk level calculation algorithm for factors in SBIR form was not obvious or easily understood - need continuous referencing to paper source to understand; limited number of health factors covered,  ***Patient level:*** Brochures and the amount of information received was overwhelming   - Existing tool did not prioritize which factors should be focused on, so nurses gave information for all medium to high risk factors. |
| **Outer Setting** | **Patients’ Needs and Resources** | **CTI:**   - ***Community level enablers needed to meet patients’ needs and resources:*** Available resources and programs in community to address SBIR factors and existing health connections – Alberta Healthy Communities program was supportive | **CTI:**  ***Patients’ needs and resources were not adequately met:***   - ***CTI did not prioritize SBIR factors:*** patients found brochures and information from SBIR overwhelming; timely resources are needed; lengthy referral wait time - ***Community’s resources for patients:*** Insufficient clinical programs or resources to support patients when they are RTC. - ***Health system barriers to meeting patient needs:*** difficult referral pathway after SBIR questionnaire was completed - long wait time reduces patients’ motivation to change; focused on disease treatment not on prevention; lack of resources to support patients’ when they are RTC - ***Patients’ knowledge and beliefs:*** patients’ health choices are based on health inequities: inability to understand scientific studies and assess risk; lack early health prevention education; lack health behavior understanding. Patients desire for immediate results from initial health choice - “an all or nothing approach” that prevents behavior change and choices; choices are limited due to poverty and convenience; lack understanding of food choices and health problems; need easy win solutions for changes to behavior - ***Relationship between provider and patients:*** lack of trust in the clinicians – past negative experience with clinicians can create hesitancy to disclose alcohol and tobacco usage |
| **Inner Setting** | **Networks and Communication** | ***IST:***   - ***Health systems level:*** Participants perceived communication was essential for SBIR implementation. The results of the collaboration, communication and coordination with other AHS teams and departments created alignment with Canadian guidelines; alignment with AHS departments on risk factor assessment; alignment with internal AHS screening guidelines; data flow from screening department - ***CTI level:*** Regular touch base conversations between CTI and IST facilitated adaptations of the SBIR; on the ground facility understanding; time to train users | ***CTI:***   - ***CTI level****:* Lack internal team communication between members about SBIR - ***Patients level:*** patients were not notified of SBIR assessment prior to appointments |
|  | **Culture** | ***IST:***  ***Health Center level:*** a culture of health promotion; hospital design and layout support health promotion | **CTI:**   - ***Healthcare Centre level barriers:*** Participants perceived innovation acceptance and value is based on evidence. Health department management were hesitant to implement SBIR due to desire for immediate results; lack culture of health promotion; need evidence to support patient outcomes – feedback reports; role of department in patient care; politics of implementation - ***CTI*** perceived that innovation culture was lacking in the hospital setting: SBIR lacked physician support, hierarchy of roles in healthcare; physicians don't learn new innovations from nurses; perceived that intervention was needed at primary care; patient's late stage of health decline; need to include intervention in primary care |
|  | **Implementation Climate** | **IST & CTI:**   - Implementation driven by managerial decision | **CTI:**   - ***Implementation climate was driven by managers:*** This was seen as a top-down process coming from managers regardless of clinical staff's own views (barriers around workflow maybe an indicator of staff hesitancy to be excited about new innovations) - ***Department hesitancy:*** patients’ late stage of health decline |
|  | **Readiness for Implementation**   1. *Available resources* 2. Access to knowledge and information 3. Leadership readiness | ***IST:***   - ***AHS health system readiness for change – leadership engagement to develop coordination readiness between depts:*** collaboration with AHS teams to align components / departments in system: align SBIR with Canadian guidelines; align with AHS departments on risk factor question: align with internal AHS screening guidelines; aligning health practices, standards, evidence, ethics.   ***Pilot site readiness:***   - ***available resources*** ***and funds needed to implement SBIR and foster buy-in:*** funding human resources; existing relationships with staff and community; meeting end-users needs (1 page format fit for clinician time; clinical - facility setting needs; workflow of clinic; motivation, enthusiasm to innovate; training provided to CTI, comprehension of SBIR - ***CTI access to knowledge and information creates confidence:*** developed scripts for CTI to reduce fear of using the tool; SBIR training for CTI; develop feedback reports for managers - ***Leadership readiness:*** Existing knowledge, skills, and leadership buy-in | **CTI:**   - ***Patient readiness to change:*** Need to consider patients' readiness for change: Ability to meet patients’ needs when they are ready - ***Community level factors impact readiness:*** External environmental factors - forest fires; existing or more resources needed to address SBIR factors; understanding community needs |
| **Characteristics of Individuals** | **Knowledge and beliefs** | Not reported | - ***CTI were hesitant to implement SBIR due to the following:*** SBIR questions to patients deemed too personal and not within provider role; small community means clinician and patient are neighbors; perception that SBIR would not make a health difference (provider see patient in late stage of health decline; provider see patients choices based on poverty and cannot make changes); patients lack interest in the questions - ***Patients’ knowledge / beliefs about health and hesitancy to change (also creates hesitancy in providers):*** desire for immediate results from initial health choice; inability to understand scientific studies and assess risk; lack early health prevention education; lack health behavior understanding - All or nothing approach that prevents behavior change and choices; choose convenience over healthy options; lack understanding of food choices and health problems - ***Pilot site management hesitancy:*** patients’ late stage of health decline and SBIR is too late |
|  | **Individual stage of change** | ***CTI level:*** willingness to participate and be involved | Not reported |
|  | **Self-efficacy** | Not reported | **CTI hesitancy:**   1. ***CTI’s lack of self-efficacy:*** 1) lacked education, skills, and training on health behavior change on risk factors since patients took an “all or nothing approach”; providers' own health choice; could not answer patients' questions on all the risk factors 2. ***Clinical workload:*** high volume of data collection; lack of time; lack of time to answer all the patients’ questions; lack time to train |
| **Process** | **Planning** | **IST:**   - ***IST planning process in AHS (systems level)***: collaboration with AHS teams to align SBIR risk factors with Canadian guidelines; align with AHS departments on risk factor question; align AHS practices, standards, evidence, ethics for risk factors; develop process to acquire data from CEPR; internal dept collaboration and coordination - ***IST planning at pilot site***: promotional material facilitate understanding - professional looking and branding facilitate buy-in - ***Planning for patients’ information needs***: printed health information easy to use; SBIR align with risk factors - ***IST operational plans needed to support SBIR implementation***: evaluation on SBIR supported by evaluation expertise; easy to understand scripts for clinical workers; training materials for users; funding a dedicated program facilitator | Not reported |
|  | **Engaging** | **IST:**   - ***IST engaging with hospital teams.*** Engagement with facilitator using regular touch base conversations facilitate adaptations of the SBIR; on the ground facility understanding; time to train users; facilitate user comfort and comprehension of the SBIR tool - knowledge and awareness risk factors; willingness of CTI to participate and be involved - ***IST facilitator engaging with CTI:*** CTI champion facilitates usage of the SBIR tool - connected other clinical staff - ***IST engagement with hospital management***: Feedback reports used to understand patient referral- Decision making around how to structure report to meet needs of hospital unit; communication and coordination for sustainability of the project; seeing the big picture or goal of the project; supportive team provided on site help | Not reported |
|  | **Executing** | **IST:**   - ***AHS systems***: SBIR form achieved systems alignment on risk factors - ***Pilot site:*** leadership support was essential to executing the implementation of SBIR: to sway CTI hesitancy to implement; top-down leadership style; good relationship between implementation lead and manager at site | **CTI:**   - ***SBIR format*** enabled user errors in data transfer; ranking was unclear for clinicians - ***Clinical knowledge gaps:*** lack of knowledge supports needed to use SBIR (toolkits of all SBIR factors; need training on how to approach behavioral change in patients); CTI found shorter training sessions needed; training needed to handle patient questions to reduce discomfort - ***Clinical workflow challenges***: competing priorities; project increased workload; not enough time for SBIR to become a work practice - ***Patients’ needs:*** patients want to be informed ahead of the appointment that they'll be completing SBIR; fit of patient appointment with topic of SBIR; trust their healthcare providers to complete SBIR; brochures, information overwhelming - ***SBIR referral process requires*** trust between different healthcare providers needed - ***Top-down leadership style:*** did not need to secure clinical staff buy-in because it was a decision by management |
|  | **Reflecting & Evaluating** | **IST:**   - ***Hospital units need for feedback reports*** were perceived as essential to pilot site management: feedback reports used to understand patient referral; provided markers of patient screened and referred; Supports managerial decision - ***Collaboration on feedback reports:*** communication and coordination in team needed; reliance on team understanding of facility management needs; Understanding each person's role in the project (including evaluation) | **IST:**   - ***Feedback reports:*** Dedicated time and expertise needed to develop reports; no data on referral to behavior change; no data on whether patient went to referral appointment; required real time completion to impact decision making; understanding needs of each unit in facility for data; high volume of data to synthesize. - ***Hospital management need for feedback reports:*** evidence needs to be timely - ***Health systems data access:*** While there was systems alignment between AHS department on SBIR factors / questions, data access was challenging: navigating internal AHS policy on data access - time consuming to sort out; ability to share information on patients across care trajectory |

SBIR: Screening brief intervention and referral

CFIR: Consolidated Framework for Implementation Research
IST: Implementation support team

AHS: Alberta Health Services

CTI: Clinical team implementers
